# The acute oncologist’s role in managing patients with cancer and other comorbidities

**DOI:** 10.15256/joc.2012.2.8

**Published:** 2012-11-05

**Authors:** Kate Shankland, Peter Kirkbride, Anne-Marie Bourke, James Price, Lucy Walkington, Sarah Danson

**Affiliations:** ^1^Department of Oncology, Weston Park Hospital, Sheffield, UK; ^2^Department of Palliative Medicine, Royal Hallamshire Hospital, Sheffield, UK; ^3^Academic Unit of Clinical Oncology, Weston Park hospital, Sheffield, UK

**Keywords:** acute oncology, acute oncologist, comorbidity, cancer

## Abstract

**Background:**

An Acute Oncology Service (AOS) is paramount to providing timely and improved pathways of care for patients who are admitted to hospital with cancer-related problems or suspected cancer.

**Objective:**

To establish an AOS pilot study to decide how best to implement such a service locally.

**Methods:**

The AOS, which included collaboration between the oncology and palliative care teams at the Northern General Hospital in Sheffield, UK, ensured that the majority of oncology patients in the region received timely assessment by an oncologist if they became acutely unwell as a result of their cancer or its treatment. The AOS consisted of a thrice-weekly ward round, and daily telephone advice service.

**Results:**

We report on patient data during the first 12 months of the pilot study. Delivery of the AOS enhanced communication between the services and provided inter-professional education and support, resulting in earlier oncological team involvement in the management of patients with cancer admitted under other teams, as well as provision of advice to patients and their caregivers and families. Provision of the AOS shortened the mean length of hospital stay by 6 days. Two case studies are presented to illustrate the typical challenges faced when managing these patients.

**Conclusions:**

Establishment of the AOS enabled effective collaboration between the oncology and other clinical teams to provide a rapid and streamlined referral pathway of patients to the AOS. Locally, this process has been supported by the development of acute oncology protocols, which are now in use across the local cancer network.

Journal of Comorbidity 2012;2:10–17

## Introduction

In the UK, the incidence of the most common cancers, including breast, lung, colorectal and prostate cancer, is strongly associated with increasing age, and, therefore, many patients who have cancer also have other comorbidities [1]. Oncologists have to tailor their treatments to account for these other health problems to optimize care for individual patients. Comorbid conditions can restrict the types of oncological intervention offered to patients, and it is important to consider the balance of benefit versus the potential risks of treatment for each patient. Chemotherapy-related toxicities, such as nausea and lethargy, may be common to the majority of agents, albeit to varying degrees. The oncology team, with the support of the palliative medicine team, manages these effects. Other side-effects are particular to specific drugs, such as the cardiac toxicity related to 5-fluorouracil (5-FU), a chemotherapeutic agent widely used in the treatment of gastrointestinal and breast cancers. The incidence of angina following treatment with 5-FU is 1.2–18.0% [2], which is relatively common given the numbers of patients receiving this drug. Serious cardiac pathology (ST-segment elevation or ventricular arrhythmias) is much less common, with an incidence of approximately 0.55% [3].

Acute oncology is a subspeciality, which has evolved relatively recently, that focuses on the prompt management of patients who are admitted to hospital because of symptoms caused by their cancer or its treatment. This includes patients for whom a new diagnosis of cancer is established following admission to hospital with symptoms that may suggest a malignant cause, such as anaemia or dysphagia. The impetus behind the creation of this subspeciality was the findings from two reports commissioned to assess the safety and quality of chemotherapy services in England; findings which highlighted the often less-than-optimal care of such patients.

In 2008, the National Confidential Enquiry into Patient Outcome and Death (NCEPOD) published the report of their study of patients who died within 30 days of receiving systemic anticancer therapies (SACT) [4]. One of the concerns raised surrounded the admission of acutely unwell oncology patients to hospitals where there are no, or limited, oncology services. In 42% of the cases reviewed, patients were admitted to a general medicine ward rather than a haemato-oncology unit after the development of complications from SACT. The report highlighted the importance of strengthening the links between oncology and general medicine to optimize the management of patients who present acutely unwell to general medical physicians with complications of SACT. The question of whether it is appropriate for such patients to be admitted under the care of general physicians when things go wrong was also raised; however, any change to current working practice would require an expansion of the oncology workforce.

Following this report, the National Chemotherapy Advisory Group (NCAG) was asked to address the concerns raised by NCEPOD. Their report, published in August 2009, recommended, amongst other things, that all hospitals with an Accident and Emergency (A&E) department establish an Acute Oncology Service (AOS), with representation from A&E, general medicine, clinical and medical oncology, haematology, oncology nursing, and oncology pharmacy [5].

In April 2011, the peer-reviewed Acute Oncology Measures were published [6]. One stipulation of the report was that all hospitals with an A&E department should establish an AOS, providing a 5-day-per-week service to enable most of the patients to be seen by an oncologist within 24 hours of admission. In an attempt to assess the local requirements for an AOS, and to inform decisions about how best to implement such a service locally, we began an AOS pilot study at the Northern General Hospital (NGH) in Sheffield, UK, in September 2010.

## Acute oncology service in Sheffield

The Weston Park Hospital (WPH) in Sheffield, UK, is one of only four specialist cancer hospitals in the country, serving a population of 1.8 million in South Yorkshire and North Derbyshire. Almost all oncology services in Sheffield, including the delivery of radiotherapy and systemic anticancer therapies, are concentrated at the purpose-built cancer centre at the WPH. The site also includes the Cancer Clinical Trials Centre where patients receive treatment in the context of clinical trials. Satellite chemotherapy units also provide a limited range of SACT in the wider region. Geographically distinct from the WPH, the NGH in Sheffield currently has 1,354 beds, and admitted 15,475 patients in the financial year ending March 31, 2011. It receives all acutely unwell adult patients in the city via the sole adult A&E department. Prior to September 2010, oncology input at the NGH was limited to one outpatient clinic and two multidisciplinary team meetings per week. There was no provision for the assessment of inpatients at the NGH by an oncologist.

In response to the NCEPOD and NCAG reports, an acute oncology pilot study was initiated in September 2010 to provide dedicated oncology input for inpatients at the NGH. For the first 7 months of the pilot study, a consultant clinical oncologist provided expertise via an acute oncology ward round for one-half day per week. This service was increased to two-and-a-half days per week in total, spread over 3 days, for the final 5 months of the pilot study by the addition of a medical oncology specialist registrar to the acute oncology team. The service was advertised repeatedly via email to all of the consultants at the NGH, with details of how to refer patients to the service.

At the same time, an AOS was also established at the WPH, where the majority of patients on anticancer therapy in the region are admitted if they become acutely unwell as a result of their cancer or its treatment. The AOS at the WPH consists of a daily consultant ward round to ensure that all newly admitted patients are seen by a consultant within 24 hours of admission, and the availability of a consultant for telephone advice for clinicians from other specialities 24 hours per day, 7 days a week.

## Results

### Analysis of the AOS data from the first 12 months of the pilot study at the NGH

**Figure 1:**
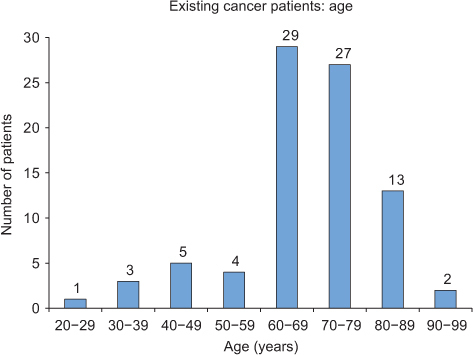
Age of existing cancer patients.

**Figure 2:**
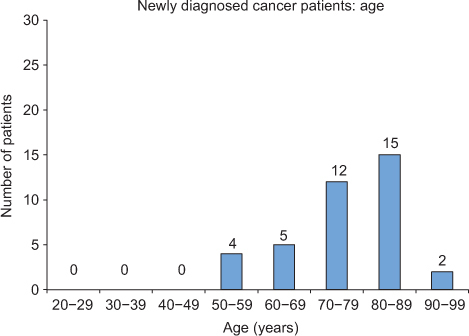
Age of newly diagnosed cancer patients.

During the first 12 months of the pilot at the NGH, 136 patients were seen. Of these, 122 sets of notes were available for analysis, although a limited amount of information was available for some of those patients for whom the notes were not available. Patient outcomes were censored on 05/09/11. Two thirds of the patients were previously known to have a diagnosis of cancer (84/136; 62%). The remainder were newly diagnosed with cancer during their admission. Figures 1 and 2 illustrate the age of the existing oncology patients and the newly diagnosed patients, respectively.

**Figure 3:**
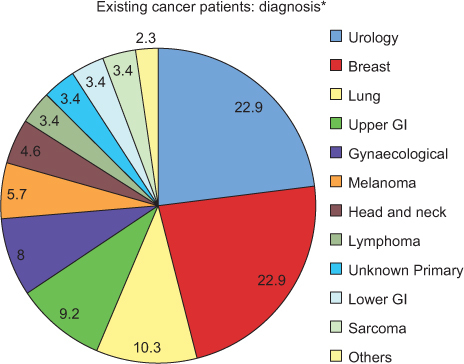
Relative frequency of the more common underlying primary tumour sites in patients with a cancer diagnosis. GI, gastrointestinal. *Three patients with two diagnoses.

**Figure 4:**
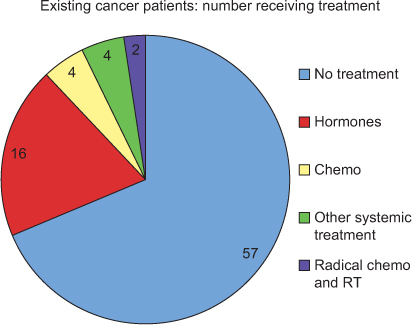
Number of existing patients receiving active anticancer treatment at the time of admission to the Northern General Hospital, Sheffield, UK. Chemo, chemotherapy; RT, radiotherapy.

Of the patients known to have a diagnosis of cancer, Figure 3 illustrates the relative frequency of the more common underlying primary tumour sites. Less than one-third of these patients (57/83) were on active anticancer treatment at the time of admission to the NGH (see Figure 4). The reason for referral to the AOS could be broadly divided into one of four categories for more than 90% of the patients, namely: investigations had revealed new findings and the admitting team required an opinion as to whether treatment was available; an opinion was sought specifically regarding palliative radiotherapy (e.g. for bone metastases); the patient had new symptoms which the admitting team thought might be due to the underlying malignancy; or the patient had been admitted with an unrelated problem, but general advice regarding the state of their underlying malignancy and/or its treatment was requested.

**Figure 5:**
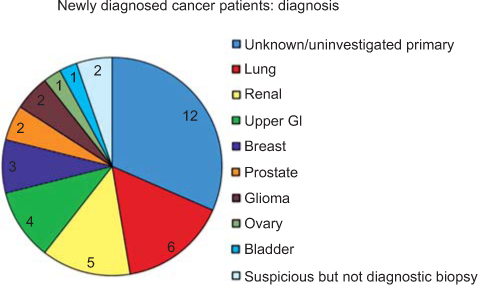
Tumour sites of newly diagnosed cancer patients. GI, gastrointestinal.

**Figure 6:**
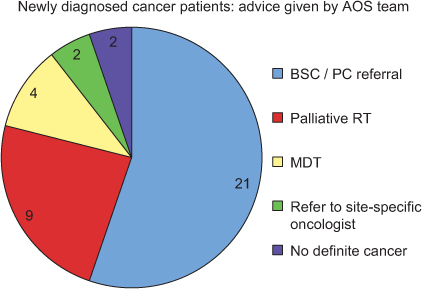
Advice given by the Acute Oncology Service (AOS) team to newly diagnosed patients. BSC, best supportive care; MDT, multi-disciplinary team meeting referral, PC palliative care, RT radiotherapy.

**Figure 7:**
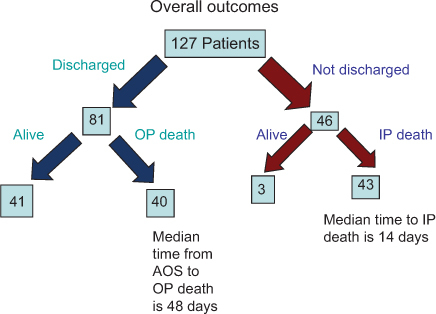
Overall outcomes of patients referred to the Acute Oncology Service (AOS) during the first 12 months (Sept 2010–2011) of the pilot study at the Northern General Hospital, Sheffield, UK. IP, inpatient; OP, outpatient.

Of the patients who received a new diagnosis of cancer during this admission, almost all (33/37; 89%) were diagnosed radiologically following investigations for new symptoms. The tumour sites involved are illustrated in Figure 5. The initial advice given by the AOS is shown in Figure 6. Just over half of these patients were unsuitable for active anticancer treatment and were therefore referred directly for best supportive care and/or palliative care. This was in part due to their poor performance status, which precluded active treatment. This was in turn influenced by their age and the presence of comorbidities. Of the remainder, most were referred for palliative radiotherapy, or referred to the site-specific multidisciplinary team or oncologist treating their respective tumour types. Of these patients, one-third ultimately went on to receive best supportive care only. Thus, overall, two-thirds of the patients who received a new diagnosis of cancer during admission to the NGH ultimately received best supportive care. Figure 7 illustrates the outcomes of the patients seen during our study. Of those patients who were discharged from hospital, half had subsequently died at the time of analysis, with a median time from contact with the AOS to death of approximately 7 weeks. Of the patients who were not discharged, the vast majority died in hospital, with a median of 2 weeks from contact with the AOS to inpatient death.

We assessed the impact of increasing the frequency of the AOS from one-half day per week to two-and-a-half days per week in terms of mean length of hospital stay (LOS). By increasing the frequency of the service, we significantly reduced the mean LOS by 6 days, from 26 to 19.8 days. The average daily income received by the trust for the patients we saw was approximately £200 per patient. A theoretical extension of their inpatient stay by 6 days would attract a much lower additional income of between £57 and £101 per night. In other words, an additional LOS by 6 days attracts little extra income for the trust, while costs continue to increase.

The two case histories described below illustrate the typical challenges we faced when contributing to the management of these patients, many of whom were receiving care from several specialist teams in addition to the oncology team.

### Case study 1

A 79-year-old man was referred to the AOS by the admitting general physicians on 14/07/11 and was seen on the same day. He was known to have metastatic malignant melanoma at the time of admission to the NGH, but was not previously under the care of an oncologist. He had been admitted on 12/07/11 with general deterioration, and had been found to have acute kidney injury (AKI) with a serum creatinine of 603 μmol/L on routine biochemistry. A computed tomography (CT) scan performed prior to this admission on 21/06/11 showed extensive pelvic nodal metastases from his melanoma, which were now presumed to have caused urinary tract obstruction resulting in AKI. Prior to the involvement of the AOS, the admitting team had sought the advice of the renal physicians as to the suitability of renal dialysis for this man. It was concluded that dialysis was not appropriate. The admitting team then requested advice from the urologists regarding the use of nephrostomies. The urologists had given advice over the telephone, as urology services are based at another hospital in the city. They had advised that nephrostomies were a possible option, but prior to insertion the patient would require re-imaging with a non-contrast CT scan to firmly establish the presumed cause of the AKI. After receiving this advice, the admitting team contacted the AOS for an opinion to help decide the best management. We discovered a patient who had a performance status of 4, who was therefore not sufficiently fit to receive any systemic treatment for his underlying melanoma, the presumed cause of his AKI. It also became clear after speaking to the patient that he did not wish to have any further intervention. Our opinion was therefore that any further investigations, and the insertion of nephrostomies, were not appropriate. We recommended that the team responsible for this man’s care discuss his medical problems and grave prognosis with the patient and his family, and instigate palliative care to manage any symptoms as appropriate. He died peacefully in hospital 6 days later.

This case illustrates some of the complexities involved in healthcare decisions, which often include opinions from several different medical and surgical specialities. Obtaining an opinion from the relevant teams can be time-consuming, and clinicians are often obliged to make assessments and give opinions without the benefit of actually seeing the patient. This can lead to them giving their “gold-standard” advice, which is often not appropriate for individual patients. It then falls to another clinician, usually the consultant under whose care the patient has been admitted, to co-ordinate the patient’s care, and decide on a management plan for their patient, having noted the advice received.

### Case study 2

A 61-year-old man not previously known to have cancer was referred to the AOS 7 days after his admission to the NGH under the care of the orthopaedic surgeons. He had been transferred from a local district general hospital for a biopsy of a vertebral lesion, which had been found on a magnetic resonance imaging scan after he presented with back pain. On arrival at the NGH, the patient had normal neurological function. Whilst the results of the biopsy were awaited, the patient developed bilateral leg weakness and sensory loss. Surgery was not thought to be appropriate. He was seen by the AOS team, treatment with radiotherapy was discussed, and the patient was transferred to the WPH later that same day for radiotherapy, which was commenced the following day. Subsequent investigations did not reveal a primary tumour, although bone biopsies confirmed metastatic adenocarcinoma. He therefore had a diagnosis of carcinoma of unknown primary. Following his radiotherapy, he was deemed too unwell for chemotherapy and received best supportive care. He died 10 weeks after his initial diagnosis.

### Oncology and palliative medicine collaborative work in Sheffield

The NGH has a palliative care hospital support team that aims to manage the symptoms of patients with potentially life-limiting conditions, including cancer. During the Sheffield AOS pilot study, the oncology and palliative care teams worked in collaboration. The main benefit was timely communication between the services; which is particularly important given the poor prognosis of this patient group. Other advantages included inter-professional education and support. Other centres in Europe have also found that despite the presence of a hospital palliative care team, oncologists require training in symptom management, as they are often involved in the end-of-life care of patients with cancer [7]. Mansour et al. took a 1-day snapshot of the inpatients at the Royal Sussex County Hospital in Brighton, UK, to investigate admissions of patients with known or suspected cancer. Of the 30 patients admitted with complications of cancer or cancer treatment, 66% were only suitable for best supportive care, yet only 7.5% of these patients had been referred to the inpatient palliative care team [8].

Despite the fact that many of the patients referred to the AOS were deemed too unwell to receive or continue SACT, they and their relatives found reassurance from a specialist oncology opinion. Those with a pre-existing diagnosis of cancer had often developed longstanding relationships with oncologists that they wanted to continue. That is, the presence of the AOS relieved psychological distress. Many patients were referred by the AOS to the palliative care team for specialist symptom control and advanced care planning. Conversely, palliative care specialists referred patients to the AOS for consideration of oncological treatment for the control of refractory symptoms, for example, radiotherapy for bone cancer pain. For those with a new diagnosis of cancer, the AOS perspective on potential anticancer treatment options and likely prognosis facilitated clinical decision-making. Finally, effective teamwork allowed accurate, consistent information to be communicated to both patients and their families.

## Discussion

The use of chemotherapy and other SACT has increased dramatically over recent years, with an increase of approximately 60% in the amount of chemotherapy delivered over a 4-year period [5]. In addition to the chemotherapeutic agents, the development of numerous targeted therapies over the last few decades has increased the choice and complexity of treatments available for many types of cancer, and in so doing, has resulted in more patients receiving more treatments for longer than was the case in the recent past. For example, systemic therapies for patients with malignant melanoma have historically been limited by low response rates. Recently, new novel treatments have emerged which have broadened the treatment choices available. In addition to the well-established dacarbazine chemotherapy, ipilimumab (a CTLA-4 inhibitor), and vemurafenib (a BRAF-kinase inhibitor), are also available. Both drugs can cause serious toxicities that may present to specialities other than oncology (see Table 1). This expansion of the choice and availability of systemic treatment also brings with it the risk of complications from treatment, which may necessitate admission to hospital, sometimes under the care of general physicians, many of whom have little or no experience of prescribing these drugs or recognizing their complications. For example, many chemotherapeutic regimens can cause neutropenia, which brings the associated risk of neutropenic sepsis. It is imperative that patients with this potentially life-threatening complication are treated immediately with intravenous broad-spectrum antibiotics to minimize the risk of multi-organ failure and death. Reported mortality rates in patients admitted with neutropenic sepsis from other centres are between 2 and 10% [9–11]. In Sheffield, all patients on SACT are advised to contact the cancer centre at the WPH directly if they become unwell. Our series of patients seen at the NGH did not include any patients admitted with neutropenic fever, which implies that local policies appear to be effective in preventing the admission of such patients to the NGH. However, patients in other series who were treated with SACT at general hospitals are often are admitted via A&E [9]. Given that SACT can have wide-ranging side-effects, which may present to various branches of medicine, one of the roles of an AOS is to provide support in the management of such patients when they are admitted under the care of non-oncologists.

In our experience, communication between teams is essential in order to minimize repetition of work and to establish more clearly the respective roles of the various clinicians contributing to the care of the patient. In the USA, sentinel events occurring in the healthcare settings are reported voluntarily, or via the complaints process, to the Joint Commission. When this occurs, the healthcare organization is required to share its root-cause analysis, which, in turn, is reviewed by a Joint Commission clinician. Breakdown in communication is one of the leading root causes of medical adverse events reported to the Joint Commission [12]. This breakdown can occur between members of the same team at shift-change, or between members of different healthcare teams, which includes the transition from primary to secondary care settings, and back again [12]. Implementing a system to ensure that at the time of discharge from secondary care settings, patients and their families, as well as the patients’ primary care physician, are provided with key information relating to recent results, diagnoses and changes in medication, can also reduce the risk of adverse events. In addition, direct communication between secondary and primary care regarding a new diagnosis of cancer in an elderly patient who is unfit for anticancer treatment, may help reduce the likelihood of readmission of such patients in the terminal phase of their illness. Open discussion about end-of-life care with these patients and their families may allow patients more autonomy in their choice regarding preferred place of death.

Our observations also show that such communication between healthcare professionals and patients is often suboptimal. Directly asking the patient about their wishes regarding further intervention is a simple, but often overlooked, way in which treatment decisions can be made collaboratively. In their document “Treatment and care towards the end of life: good practice in decision making”, the General Medical Council states that doctors should, “Respect patients’ right to reach decisions with you about their treatment and care” [13]. During our AOS pilot study, our experience has been that many elderly patients who are approaching their end-of-life are aware of that fact, and are not distressed when asked to discuss their wishes regarding end-of-life care. As oncologists, we perhaps have more experience of such discussions with our patients than do doctors from some other specialities. However, it is the responsibility of all doctors to address such issues with patients and their families when necessary, as it gives patients some autonomy at a time when treatment options are often limited.

Our experience shows that patients who have cancer and require emergency admission to hospital, or who have symptoms requiring admission to hospital – which are subsequently found to be due to cancer – tend to be elderly, and have poor outcomes. Prior to the AOS pilot study in Sheffield, individual oncologists were in direct communication with a limited range of clinicians from other specialities when attending tumour site-specific multidisciplinary team meetings. During the pilot study, we have received referrals from numerous sources, necessitating clear communication between clinical teams who did not previously have established links. The AOS provides a platform from which non-oncologists can be informed about the toxicities of SACT, thus encouraging a more streamlined referral pathway of such patients to the AOS. Locally, this process has been supported by the development of acute oncology protocols, which are now in use across the local cancer network.

In addition, patients were referred to us who had a newly diagnosed, often un-investigated primary, when historically, oncologists would only have received referrals of patients who have undergone the traditional “work-up”, including radiological and histological assessment. Our data show that the majority of these patients are elderly, and have comorbidities, and few go on to receive any specific oncological intervention. By becoming involved in the care of such patients at a much earlier stage than would traditionally have been the case, we have been able to help direct their management, which in two-thirds of cases was towards best supportive care. Thus, we hope to have avoided unnecessary investigations for patients who have a poor performance status and are therefore not sufficiently fit to receive anticancer therapy. King and Leonard report similar findings from The Whittington Hospital in London, UK, where the introduction of an AOS resulted in fewer blood tests, biopsies and endoscopies, and also reduced the LOS in these patients [14, 15]. By consulting with patients and their families, we have established their wishes, and have hopefully provided them with some autonomy as they approach their end-of-life. In addition to these clinical benefits, our data show that the provision of an AOS led to a reduction in the mean LOS of these patients. This, along with the savings associated with a reduction in unnecessary investigations in patients who are too unwell for active anticancer treatment, is an important consideration in the current financial climate.

## Figures and Tables

**Table 1:** Examples of novel anticancer agents, their indications and toxicities.

Drug	Class	Indications	Toxicities
Bevacizumab (Avastin^®^)	mAb	Metastatic breast cancer, metastatic colorectal cancer, non-small-cell lung cancer, glioblastoma multiforme, metastatic renal cell cancer	Haemorrhage, gastrointestinal perforation, delayed wound healing, thromboembolism, hypertension, proteinuria
Cetuximab (Erbitux^®^)	mAb	Metastatic colorectal cancer, head and neck cancer	Infusion reactions, acneiform rash, nausea, diarrhoea, hair changes, sore eyes
Erlotinib (Tarceva^®^)	EGFR, TKI	Non-small-cell lung cancer	Rash, diarrhoea, fatigue
Gefitinib (Iressa™)	EGFR, TKI	Non-small-cell lung cancer	Rash, diarrhoea, stomatitis, fatigue
Ipilimumab (Yervoy™)	mAb	Advanced melanoma	Immune-mediated colitis, hepatitis, toxic epidermal necrolysis, neurological sequelae
Rituximab (MabThera^®^)	mAb	Non-Hodgkin lymphoma,chronic lymphocytic leukaemia	Severe cytokine release syndrome, haematological toxicities, cardiac toxicities
Trastuzumab (Herceptin^®^)	mAb	Breast cancer, metastatic gastric cancer	Heart failure, infusion reactions

EGFR, epidermal growth factor receptor; TKI, tyrosine kinase inhibitor; mAb, monoclonal antibody.
